# Interaction of Lifestyle and Work Ability Index in Blue Collar Workers

**DOI:** 10.5539/gjhs.v7n3p90

**Published:** 2014-11-17

**Authors:** Saber Mohammadi, Mostafa Ghaffari, Alireza Abdi, Baharak Bahadori, Elham Mirzamohammadi, Mirsaeed Attarchi

**Affiliations:** 1 Occupational Medicine Department, Faculty of Medicine- Iran University of Medical Sciences, Tehran, Iran; 2 Brain and Spinal Injury Research Center (BASIR), Imam Khomeini Hospital, Tehran University of Medical Sciences, Tehran, Iran

**Keywords:** lifestyle, work ability index

## Abstract

**Background:**

Early labor force exit is one of the major problems worldwide. The present study investigates the relationship between work ability and lifestyle.

**Methods:**

This study was conducted at a manufacturing plant in Tehran in 2012. All 851 male workers in this plant were included into the study and their work ability was assessed using the Work Ability Index (WAI). Based on the obtained scores, the participants were then classified into four work ability groups (poor, moderate, good, or excellent). Moreover, the participants’ lifestyles were studied in three areas, including physical activity, cigarette smoking, and Body Mass Index (BMI).

**Results:**

The average work ability index score was 42.07, ranging from 7–48. Among the participants, 6.4% (43), 6.5% (44), 38.3% (259), and 48.8% (330) were in the groups with poor, moderate, good, and excellent work ability, respectively. The results of logistic regression analysis showed that there is a significant relationship between work ability and lifestyle (cigarette smoking, BMI, and physical activity) even after adjustment for confounding variables (P<0.05).

**Conclusions:**

According to the results of the present study, there might be a relationship between work ability and lifestyle (physical activity, BMI, cigarette smoking). Therefore, it is recommended to implement a lifestyle quality enhancement program to improve work ability in working environments.

## 1. Introduction

Early labor force exit from working environment is one of the major problems in developed and developing countries (Ilmarinen, 2001). One reason for this issue is that the young workforces enter working environments with delay due to long-term education. Moreover, given that the global population (including Iran) is ageing since the first half of the 21st Century, a series of problems are predictable to occur for industries and working environments (Kumashiro et al., 2005; Mehryar & Ahmad-Nia, 2004). Some of these problems include reduced young workforce, increased pressure on old workers, and probably increased frequency of disease among these workers, and more importantly, their reduced work ability and early exit. Therefore, assessment of work ability and identification and prediction of the people with poor work ability and performance in future have particular importance.

Work ability means how much a worker can work now and in the near future; and that – given the job demands, personal health status, and mental resources–how he/she will do his work (Lin et al., 2006).

The Work Ability Index (WAI) is a tool used for research purposes and clinical assessments in the field of occupational health to assess work ability during occupational health examinations and work environment monitoring (Ilmarinen, 2007). By means of this tool, the workers at risk of work-related disabilities can be identified. Moreover, this index helps identify the workers at risk of using long-term sick leave and early exit from work. According to the past studies, the main factors affecting ability of work include job factors, lifestyle, and health status.

On the other hand, lack of leisure-time physical activity is considered as a major health challenge. In Canada in 1999, 2.1 billion dollars or 2.5% of all the total direct health care costs was attributed to lack of physical activity (Katzmarzyk et al., 2000). Previous studies have investigated the relationship between leisure-time physical activity and some occupational factors as well as work ability. In Iran, however, few studies have been conducted on the level of physical activity among workers and its relationship with work ability.

In a study in Canada (2000-2001), Smith and Morassaei (2011) determined the relationship between leisure-time physical activity and workplace demands. In a study on the 17- 64-year-old workers (2005), Kouvonen et al. (2005) determined the relationship between leisure-time physical activity and workplace stress. In a study on 187 full-time workers in Poland (2001-2003), Kaleta et al. (2006) observed a significant positive relationship between lifestyle (not smoking cigarette, appropriate weight, proper nutrition, and regular physical activity) and WAI. In a study on the 40 -60-year-old residents of Helsinki, Lahti et al. (2010) reported the relationship between physical activity, reduced work absence and probably its role in improving work ability.

In a study on 403 firefighters, Airila et al. (2012) determined the relationship between WAI and age, lifestyle, occupational factors, and physical activity. In another study, Kaleta et al. (2004) observed a significant relationship between work ability and leisure-time physical activity. Moreover, in a study by Ranta and Pohjonen (2001), it was found that physical activity in the workplace prevents early exit from work by improving physical capacity. The results of a study by Mackey et al. (2007) on workers over 45, which was conducted through a 12-week planning for physical activity in the workplace, confirm the results of the study mentioned above.

In the present study, the researcher investigates the relationship between the level of work ability based on the Work Ability Index questionnaire and lifestyle at a manufacturing plant.

## 2. Materials and Methods

The present study was conducted at a manufacturing plant in Tehran in 2012. All 851 male workers in this plant were included into the study. The participants’ demographic, medical, and occupational information was collected using a direct interview. Among 851 workers at production line, 676 consented to cooperate and participated in our study (Response Rate: 79.43%). The present study investigates the participants’ lifestyle in three areas, including physical activity, cigarette smoking, and BMI. The participants’ weight (based on Kilogram) and height (based on meter) were measured, and the BMI (Body Mass Index) was calculated in kg/m^2^ (kilograms per square meter). They participated voluntarily in this study and signed informed consent form. This study was approved by the ethics committee of Iran University of medical sciences.

The participants’ work ability was measured using a standard questionnaire named Work Ability Index (Van den Berg et al., 2008). The reliability and validity of Persian version of the WAI questionnaire have been identified by another study (Abdolalizadeh et al., 2012). Based on Abdolalizadeh et al study the WAI questionnaire has an acceptable validity and reliability (Cronbach’s alpha coefficient: 0.77). The questionnaire consists of 7 items, each with a variable rating system. The first item is “ongoing ability of work compared with the lifetime best’, ranging from 0 to 10 scores. The second item is “work ability in relation to job demands”, ranging from 2 to 10 scores. The third item is “number of current diseases diagnosed by a physician”, ranging from 1 to 7 scores. The fourth item is “estimated work impairment due to diseases”, ranging from 1 to 6 scores. The fifth item is “sick leave during the past year”, ranging from 1 to 5 scores. The sixth item is “prognosis of work ability within 2 recent years”, with 1, 4, or 7 scores. Eventually, the seventh item is “mental resources”, ranging from 1 to 4 scores. In all above mentioned items, the highest scores belong to workers with best status of workability. The WAI is computed by the sum of the scores of all 7 items mentioned above. Higher scores of WAI are expressive of further ability of work in participants. Total score of WAI ranging from 7 up to 49. In addition, the workers were classified into four groups of work ability, including poor group (score range: 7-27), moderate group (score range: 28-36), good group (score range: 37-43), or excellent group (score range: 44-49) (Van den Berg et al., 2008).

The participants’ physical activity level was assessed using the Persian version of the short form of IPAQ (International Physical Activity Questionnaire) via a direct interview, (Hazavehei et al., 2009). The questionnaire consisted of 7 questions, and it was interpreted and scored based on the IPAQ scoring protocol (IPAQ, 2010). Physical activity is assessed by IPAQ in four areas, including leisure-time activity, domestic and gardening (yard) activities, work-related physical activity, and transport-related physical activity. The IPAQ-short form revolves around 4 types of specific activities in the 4 areas mentioned earlier. Different types of the activities assessed include sitting, walking, moderate-intensity activities, and vigorous-intensity activities occurring for at least 10 minutes during the past 7 days.

The items of the IPAQ-short form are scored separately for walking, moderate-intensity activities, and vigorous-intensity activities. In the calculations, the physical activity data were converted to min/week index, and then explained by the Metabolic Equivalent of Task (MET-minutes/week) and based on the IPAQ guide. In this questionnaire, the following calculations are used to calculate the total physical activities during the past week:

The amount of energy consumed for walking by MET×min/week is calculated based on the following method: 3.3 multiply by “walking time by minutes per day” multiply by “number of walking days per week”.

The amount of energy consumed for a moderate-intensity activity by MET×min/week is calculated based on the following method: 4.0 multiply by “moderate-intensity activity time by minutes per day” multiply by “number of moderate-intensity activity days per week”.

The amount of energy consumed for a vigorous-intensity activity by MET×min/week is calculated based on the following method: 8.0 multiply by “vigorous-intensity activity time by minutes per day” multiply by “number of vigorous-intensity activity days per week”.

The total physical activities during the past week are calculated by the following method: “the amount of energy consumed for walking” plus “the amount of energy consumed for moderate-intensity activity” plus “the amount of energy consumed for vigorous-intensity activity” during the past week, which is reported by MET×min/week.

Moreover, based on their questionnaire scores, the participants were classified into 3 groups, including low, moderate-intensity, and vigorous-intensity physical activities. Those with energy consumption lower than 600 MET, 600-3000 MET, and more than 3000 MET were included in the low physical activity, moderate-intensity activity, and vigorous-intensity activity groups, respectively (IPAQ, 2010).

For quantitative variables, Mean, standard deviation (SD) or range were computed. For comparing quantitative variables among the groups we used the independent-samples T-test.

For comparing qualitative variables the Chi-square test was performed. Logistic regression analysis was use for recognition of risk of moderate or lower ability of work in participants.

Also to investigate the correlation between ability of work and lifestyle in participants, Logistic regression analysis was performed. The significance level of two-sided p value was considered as 0.05. We used odds ratio (OR) with 95% confidence intervals (95% CI) for showing the results of statistical analysis. SPSS version 11 software was used for computation of all statistical analysis.

## 3. Results

The present study investigates 676 male production line personnel of an automobile manufacturing plant. The average age of the participants was 34.74, ranging from 21 to 63; and the average work experience was 7.34, ranging from 1 to 40.

The average WAI was 42.07, ranging from 7 to 48. In terms of WAI, 43 (6.4%), 44 (6.5%), 259 (38.3%), and 330 (48.8%) were in the poor, moderate, good, and excellent groups, respectively. Therefore, 12.9% of the participants were in the poor or moderate groups, and 87.1% of them were in the good or excellent groups. The average score of different WAI items is shown in Table 1.

**Table 1:** The Work Ability Index (WAI) items

Item	Mean of score	SD	Min-Max
1-Current work ability compared with the lifetime best	8.99	1.32	0–10
2-Work ability in relation to the demands of the job	8.80	0.78	2-9
3-Number of current diseases diagnosed by a physician	5.70	1.51	1–7
4-Estimated work impairment due to diseases	5.59	0.76	1–6
5-Sick leave during the past year (12 months)	4.52	0.68	1–5
6-Prognosis of work ability within 2 recent years	6.44	0.52	1–7
7-Mental resources	2.01	0.99	1–4

**Total score**	**42.07**	**6.04**	**7-48**

The lowest average belongs to mental resources (2.01), which is about 50% of the maximum predicted score.

For different lifestyle dimensions, the average level of physical activity was 2646.89, ranging from 0 to 18396 MET×min/week. From among the participants, 261 (38.6%), 206 (30.5%), and 209 (30.9%) had low, moderate-intensity, and vigorous-intensity physical activities, respectively.

The participants’ average BMI was 26.31 kg/m^2^, and the BMI was less than 25 kg/m^2^ for 283 (41.9%) of them, while for 393 (58.1%), it was greater than or equal to 25 kg/m^2^. Moreover, 153 (22.6%) were cigarette smokers.

Table 2 compares the average WAI in terms of the BMI, physical activity, and smoking cigarette.

**Table 2:** Comparison of the mean of work ability index in terms of body mass index, physical activity and smoking

Variable	WAI			

	Status	Mean	S.D	P-value
**Body mass index** (kg/m^2^)	≥ 25 (n= 393)	41.65	6.72	0.031
	< 25 (n= 283)	42.66	4.88	

**Smoking**	Yes (n= 153)	40.27	6.80	< 0.001
	No (n= 523)	42.60	5.71	

**Physical activity** (MET.min/week)	Low (≤ 600) (n= 261)	40.63	6.71	< 0.001
	Moderate(600-3000)(n= 206)	42.53	5.47	
	High (> 3000) (n= 209)	43.42	5.28	

As the results show, the average WAI in the group with a BMI score less than 25 kg/m^2^, the non-smoker group, and the group with vigorous-intensity activity was significantly higher than the group with a BMI score greater than or equal to 25 kg/m^2^, the smoker group, and the group with moderate-intensity activity, respectively (P<0.05).

Table 3 compares the frequency of the study population in 2 groups with poor or moderate, and good or excellent work ability in terms of the BMI, physical activity, and cigarette smoking.

**Table 3:** Comparison of the frequency of participant in two groups of work ability index in terms of body mass index, physical activity and smoking

WAI	Body mass index N-(%)		Smoking N-(%)		Physical activity N-(%)		

	≥25 kg/m^2^ (n= 393)	<25 kg/m^2^ (n= 283)	Yes (n= 153)	No (n= 523)	Low (n= 261)	Moderate (n= 206)	High (n= 209)
**Group 1 (n=87) (poor or moderate)**	67(17)	20 (5.1)	34 (22.2)	53 (10.1)	55 (21.0)	20 (9.7)	12 (5.8)

**Group 2 (n=589) (good or excellent)**	326 (83)	263 (94.9)	119 (77.8)	470(89.9)	206 (79.0)	186 (90.3)	197(94.2)
**O.R**	2.70		2.53		-----------		

**95% C.I**	1.59- 4.56		1.57- 4.07		-----------		

**P-value**	< 0.001		< 0.001		< 0.001		

The frequency of those with good or excellent work ability was significantly higher than those with poor or moderate work ability (in the group with a BMI score less than 25 kg/m^2^ compared to the group with a BMI score greater than or equal to 25 kg/m^2^, the non-smoker group compared to smoker group, and the group with vigorous-intensity activity compared to the group with low or moderate-intensity activity) (P<0.05).

For a closer look at the relationship between work ability and lifestyle, logistic regression analysis was used. In this analysis work ability was considered as a dependent variable, and the participants were classified into two groups (poor or moderate work ability and good or excellent work ability) based on the scoring of the WAI questionnaire. Lifestyle in three dimensions (cigarette smoking, BMI, and physical activity) was assessed using the IPAQ. The variables of age, marital status, level of education, physical work environment factors (assessed by the MUSIC-Norrtalje Questionnaire), and psychosocial factors (assessed by the Copenhagen Psychosocial Questionnaire) were considered as the confounding variables.

The results of this analysis showed that there is a significant relationship between work ability and lifestyle (cigarette smoking, BMI, and physical activity) even after adjustment for confounding variables (P<0.05). It was also shown that as the participants’ level of physical activity increases from poor to vigorous-intensity, their work ability increases as well (Table 4).

**Table 4:** Relationship between work ability index and life style by logistic regression analysis

Variable	Status	Adjusted OR	95% C.I	P-value
**Body mass index** (kg/m^2^)	< 25	1.00	-----------	-------
	≥ 25	2.32	1.12-4.80	0.023

**Smoking**	No	1.00	-----------	-------
	Yes	2.80	1.39-5.64	0.004

**Physical activity**	High	1.00	-----------	-------
	Moderate	3.21	1.48-6.97	0.003
	Low	4.59	1.80-11.68	0.001

## 4. Discussion

Study and estimation of WAI in working environments are very valuable for the purpose of planning and health promotion of human resources. Awareness of this index enables us to identify the workers at risk of work-related disability. The index, in fact, helps identify the workers at risk of using long-term sick leave and early exit from work.

In the present study, the average WAI was 42.07; and 48.8%, 38.3%, and 6.5% of the participants were in the groups with excellent, good, and moderate WAI, respectively.

Kaleta el al. (2010) conducted a study on 94 male and 93 female workers in Poland to investigate the relationship between the WAI and lifestyle. They found that 38.3%, 46.8%, and 14.9% of the male workers were in the groups with excellent, good, and moderate WAI, respectively. In the present study, the average WAI was 41.9% for the male population.

The level of work ability in the present study, compared to the results obtained from the study of Ilmarinen (1999), was in a more unfavorable situation. The latter, which was conducted on Western European workers, showed that 71.9% of the workers had excellent work ability. These results indicate that the WAI score is lower in Iranian workers than in Western European workers, closer to Eastern European workers such as in Poland.

About the item of mental resources in the present study, 50% of the participants obtained a lower score than the maximum predicted score, which further attracts our attention towards supporting and improving workers’ psychological health.

The results of the present study showed that there is a significant relationship between WAI and lifestyle (cigarette smoking, BMI, and physical activity) even after adjustment for confounding variables (P<0.05). This relationship has been confirmed in some studies (Fischer et al., 2006; Lusa et al., 2002; Tuomi et al., 2004). Moreover, in the study of Kaleta et al., (2006) there was an association between work ability and lifestyle. For the males with inappropriate lifestyles, compared to those with appropriate lifestyles, the risk of poor to moderate work ability was about 7 times higher. In this study, there was a significant association between WAI and cigarette smoking, BMI, and physical activity. The researchers of the present study have found that enhancing lifestyle is effective in increasing work ability. Other studies have also determined the relationship between lifestyle and work ability to some extent (Tuomi et al., 1997). Moreover, a study has shown that increased leisure-time physical activity is effective in increasing the WAI (Ilmarinen, 1999).

In the present study, for those with a BMI of 25 kg/m^2^, the risk of poor and moderate WAI was 2.2 times significantly greater than those with a lower BMI. The results of the study by Kaleta et al. (2006) showed that for women, the risk of poor or moderate WAI in the group with overweight was 2 times greater than in those with an appropriate weight (adjusted OR = 2.33; 95% CI: 1.09–7.96). In males, however, this relationship was not statistically significant.

In the present study, the risk of poor or moderate WAI for the smokers was 2.8 times significantly greater than the non-smokers. In the study of Kaleta et al. (2006), a significant relationship was observed between cigarette smoking and poor WAI among females (adjusted OR = 14.84; 95% CI: 3.07–26.42). In male cigarette smokers, the risk of poor WAI was 2 times greater. However, this relationship was not significant. According to the World Health Organization (1997), there is a negative relationship between cigarette smoking and health status, which can affect the WAI.

According to some studies, exercise at work can enhance work ability (Nurminen et al., 2002). Previous studies show the positive effect of exercise at work on the WAI (Mackey et al., 2007).

In the present study, for the people with low and moderate -intensity physical activity, the risk of poor and moderate WAI was 4.5 and 3.2 times significantly greater than those with vigorous-intensity activity, respectively.

In their study on 198 professional workers, Kaleta et al. (2004) investigate the relationship between leisure-time physical activity and the WAI. They used the Seven Day Physical Activity Recall (SDPAR) to assess physical activity. They also observed a significant relationship between leisure-time physical activity and the WAI (r=0.3, P<0.0001), and recommended that increased leisure-time physical activity could enhance work ability. In their study, the level of leisure-time physical activity for 2% of the participants was more than 2000 kcal per week, while in the study of Makowiec-Dabrowska et al. (2000), 20.6% of the participants had the same level of leisure-time physical activity. Moreover, in the study of Kaleta et al. (2003), 15.4% of the participants had the same level of leisure-time physical activity.

This difference could be due to cultural differences, level of education, and measuring tools.

Difference of lifestyles in different societies could be due to differences in cultures, societal values, and promotion of education on the proper way of living in different societies.

Being a cross-sectional study is one of the limitations of the present study, which makes it insufficient to conclusively prove a causal relationship. Therefore, it is recommended to conduct a longitudinal study to investigate the relationship between lifestyle and work ability.

The present study showed that there might be a relationship between work ability and lifestyle (physical activity, BMI, cigarette smoking). Therefore, it is recommended to implement educational programs on quitting or prevention of cigarette smoking, having a healthy diet, and doing appropriate sporting activities in order to enhance workers’ work ability and occupational health quality in working environments.
